# Myocardial Involvement in Catastrophic Antiphospholipid Syndrome during Pregnancy or Puerperium: A Case of a Young Breastfeeding Woman and Literature Review

**DOI:** 10.3390/jcm13164732

**Published:** 2024-08-12

**Authors:** Leonardo Varotto, Luca Spigolon, Alberto Dotto, Denis Leonardi, Giulia Bragantini, Luca Felice Cerrito, Cristina Deluca, Ariela Hoxha

**Affiliations:** 1 Department of Cardiology, San Bortolo Hospital, viale Rodolfi 37, 36100 Vicenza, Italy; giulia.bragantini@aulss8.veneto.it (G.B.); luca.felice.cerrito@gmail.com (L.F.C.); 2 Department of Radiology, San Bortolo Hospital, Viale Rodolfi 37, 36100 Vicenza, Italy; lucaspigolon@gmail.com; 3 Department of Medicine—Division of Cardiology, University of Verona, Piazzale A. Stefani 1, 37126 Verona, Italy; albertodotto94@gmail.com (A.D.); leonardidenis@gmail.com (D.L.); 4 Department of Neurology, San Bortolo Hospital, Viale Rodolfi 37, 36100 Vicenza, Italy; cristina.deluca@aulss8.veneto.it; 5 Internal Medicine Unit, Thrombotic and Hemorrhagic Diseases Unit, Department of Medicine, University of Padua, via Giustiniani 2, 35128 Padova, Italy; ariela.hoxha@unipd.it

**Keywords:** catastrophic antiphospholipid syndrome, antiphospholipid syndrome, pregnancy, heart involvement, myocardial infarction with non-obstructive coronary arteries, cardiac magnetic resonance imaging

## Abstract

Catastrophic Antiphospholipid Syndrome (CAPS) is a rare complication that can occur in patients with Antiphospholipid Syndrome (APS). CAPS occurs even more rarely during pregnancy/puerperium and pregnant patients, even less likely to show cardiac involvement without signs of damage on ultrasound and angiography with non-obstructive coronary arteries. We present a case of a 26-year-old breastfeeding woman, the youngest described with CAPS and acute myocardial infarction, whose diagnosis was made with cardiac magnetic resonance imaging (CMRI). A literature review of pregnant patients with similar problems was performed. There are diagnostic and therapeutic difficulties in treating these patients. CMRI demonstrated a transmural late enhancement area. A combination of therapies led to rapid clinical improvement. CMRI is an underused tool that reaffirms the pathophysiology of CAPS and leads clinicians to the possibility of a diffuse thrombotic process. CAPS involves more organs with high mortality rates. CMRI could be optimized in order to reach an early diagnosis and the most effective treatment. This study provides real-world evidence of the feasibility of MRI in a primary care setting during pregnancy/puerperium. Evidence from this study may influence future APS screening and inform policymakers regarding the use of leading MRI technology in the detection of the thrombotic process in a primary care setting.

## 1. Introduction

Anti-phospholipid antibodies (aPLs) are the serological biomarkers of anti-phospholipid syndrome (APS), an autoimmune disease characterized clinically by vascular events (arterial, venous or capillary thrombosis) and/or pregnancy mortality. One of the rarest and most serious forms of APS is catastrophic APS (CAPS). CAPS is defined as multiorgan thrombosis, affecting at least three different organs, requiring histopathological confirmation of small vessel occlusion in a minimum of one organ or tissue, and the presence of antiphospholipid antibodies at two separate moments at least 6 weeks apart (not necessarily at the time of the event) [1,2] (Table 1).

The incidence of CAPS is very rare and has been reported in 0.8% of patients with APS [1]. The most common cardiac presentation in APS is valvular involvement, specifically nonbacterial thrombotic endocarditis [4]. Nevertheless, the data from the latest review of cardiac involvement in CAPS report that the main cardiac manifestations were heart failure (55%), valve disease (31%) and acute myocardial infarction (28%) [5]. A hypercoagulable state, which confers protection against hemorrhagic challenges such as childbirth or miscarriage, can present itself in pregnancy. There have been very few case reports of myocardial infarction in CAPS, and even rarer cases in pregnant or puerperal patients.

The imaging techniques used in these young patients often do not correspond with the first clinical symptoms, and, above all, cardiopulmonary involvement is not always detectable. The challenge is to understand how to identify CAPS and its potentially lethal manifestations in these patients, considering early and effective diagnostic/therapeutic management. 

Here, we describe an extremely rare and potentially lethal condition, resolved with a multidisciplinary approach with the help of cardiac magnetic resonance imaging (CMRI) and targeted therapy. We also bring to attention the literature review of a similar case with cardiac involvement.

## 2. Case Presentation

A 26-year–old woman affected by APS with previous deep vein thrombosis and triple aPLs positive title was admitted to our department of cardiology five days after labor induction due to intrahepatic cholestasis of pregnancy (37^+3^ weeks pregnant, Gravida 1, Para 0000). The baby was healthy (2835 g, Apgar 9/10) and there was no need for neonatal intensive care units. As soon as the pregnancy test was positive, the patient replaced warfarin with subcutaneous enoxaparin 6000 UI per day. During the last month of pregnancy, she developed mild hypothyroidism, which was not treated with any therapy. In emergency care, she was symptomatic for intermittent chest-epigastric pain, with dyspnea and fever. Any other common cardiovascular risk factors were excluded; livedo reticularis was previously detected during pregnancy. She was in anticoagulant therapy with enoxaparin at 4000 UI per day. APLs included Lupus Anticoagulant, IgG and IgM anti-Cardiolipin (aCL) and anti-β2glycoprotein (aβ2GPI). Small placental infarcts appeared at histology.

By electrocardiogram (ECG), the rhythm was determined to be sinus, without evidence of inappropriate ST segment or T wave morphologies. Baseline laboratory tests (Table 2) revealed an elevation of I-Troponin (TnI) with an initial plateau, an increase in C-reactive protein and D-dimer, low hemoglobin levels, a low platelet count and antinuclear antibodies (ANA) 1:160 (SSB/La). The platelet count was 50% lower than her previous result (186,000/μL). 

**Figure 1:**
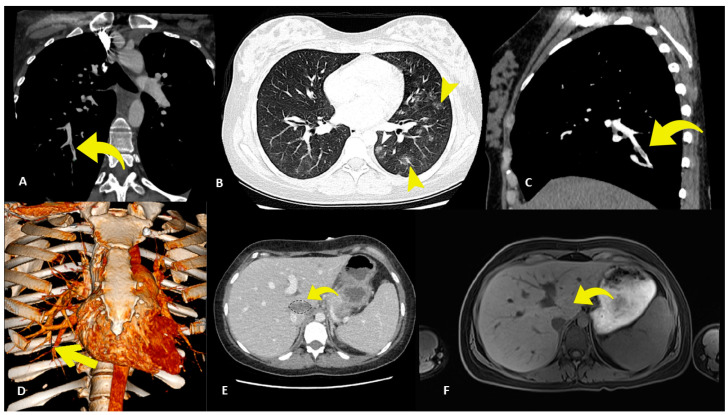
(**A**) Angiography CT scan showing the presence of some filling defects (yellow curved arrow) in a segmental branch of the right lower lobe of the lung. (**B**) Lung CT scan with axial scanning showing the presence of multiple ground-glass opacities in the lower lobes (small yellow arrowhead). (**C**) Multiplanar reconstruction of angiography CT scan highlighting some filling defects (yellow curved arrow) in a segmental branch of the right lower lobe of the lung. (**D**) Volume-rendering reconstruction highlighting the location of a filling defect in a branch of the right lower pulmonary artery (yellow straight arrow). (**E**) Contrast-enhanced CT image revealing a hypodense formation anterior to the inferior vena cava. (**F**) MRI image demonstrating disappearance and healing of the hepatic lesion.

Echocardiography showed normal left and right systolic ventricular function, with a small pericardial echo-free space suggestive of pericardial effusion. Angiography-Computed Tomography (Angio-CT) revealed a small thromboembolic event and ground-glass lungs compatible with hemorrhagic alveolitis (Figure 1A–D). The test for the detection of Severe Acute Respiratory Syndrome-Coronavirus-2 was negative.

The anticoagulant therapy had been optimized with enoxaparin 6000 UI twice a day, combined with antiplatelet therapy and acetyl-salicylic acid (ASA) 100 mg per day.

After one week of intermittent fever, the patient suffered from increased epigastric pain, diffuse headache and vomit attacks with blood clots, with drowsiness up to a comatose state. Cerebral CT and MRI excluded acute thrombotic or hemorrhagic events. Total-body CT was negative, except for minimal effusions in the pericardium, gallbladder, peri-splenic space and recto-uterine pouch; contrast-enhanced CT highlighted a hepatic hypodense formation anterior to the inferior vena cava (Figure 1E).

The patient underwent four cycles of plasmapheresis and methylprednisolone 50 mg three times a day for the first three days, with clinical improvement in our Cardiac Intensive Care Unit. Antibiotic prophylaxis with cefazolin was introduced. For the increase in TnI up to 4741 ng/L, the patient underwent coronary angiography, showing non-obstructive coronary arteries. 

**Figure 2:**
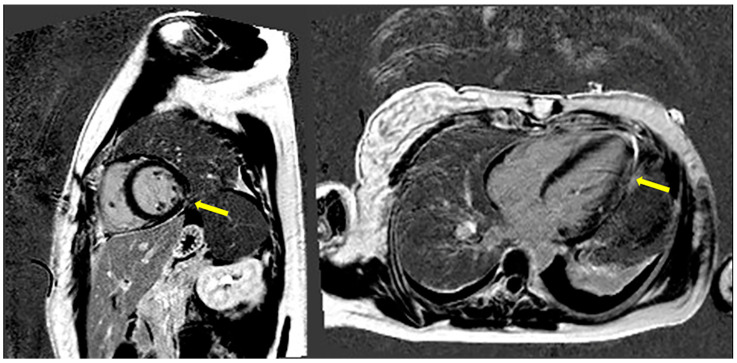
CMRI PSIR T1 sequences. Evidence of LGE suggestive of focused transmural myocardial fibrosis affecting the middle lateral left ventricle wall, through cardiac two-chamber short axis (**on the left**) and four-chamber long axis (**on the right**).

CMRI was performed, revealing by phase-sensitive inversion recovery (PSIR) T1 a small transmural late gadolinium enhancement (LGE) area on the middle lateral left ventricle wall (Figure 2).

No bright signs of myocardial edema by T2, short tau inversion recovery (STIR) or PSIR T1 (with early gadolinium enhancement—EGE) were detected. The first pass of contrast by PSIR T1 was negative for epicardial coronary ischemic events. 

On the third day after the event, one bolus of Antithrombin III 1000 UI was given; corticosteroid was reduced to 40 mg per day; and intravenous human IgG 400 mg pro kg per day (30 gr per day) was administered for 5 successive days. The patient was also transfused with one red blood cell bag.

Finally, the patient clinically improved without ventricular dysfunction or neurological residual deficit; TnI decreased to 509 ng/L and the platelet count was recovered (242,000/µL). A follow-up MRI showed the disappearance and healing of the liver lesion in the paracaval location (Figure 1F).

Discharge therapy included Enoxaparin 6000 IU twice a day, ASA 100 mg per day, Prednisone 30 mg per day and gastro-protection. At 24-month follow-up, the patient was regularly taking warfarin for APS, at the International Normalized Ratio (INR) 2.5–3.5 in good time relating to the therapeutic range (TTR) >65%. She performed follow-up laboratorial tests, with hemoglobin, platelets and troponin I always within the normal range. However, the aPLs values were confirmed to be positive. 

## 3. Literature Review

**APS.** The occurrence of acute myocardial infarction (AMI) in APS has been known since the syndrome was first described [6].

Accelerated atherosclerosis of the coronary arteries, as well as microvascular injury or coronary thromboembolism, may lead to ischemia and may be the first sign of APS [7]. About 2.8–5.5% of patients with APS may develop AMI due to atherosclerosis or, especially in young people, coronary thromboembolism [1,8].

A strong correlation between thrombophilic conditions and AMI in APS patients exists, but not for other thrombophilic disorders [9].

The association between aPLs and AMI is more frequent in women and usually occurs in the fourth decade of life [1,10]. However, AMI in younger people is generally associated with normal or almost normal coronary angiographic studies [1,11].

In the Euro-phospholipid cohort, which included the largest series of 1000 APS patients, there were significantly more AMI events in the APS-systemic lupus erythematosus (SLE) group (3.8%) compared to the primary APS group (1.2%) over a 10-year follow-up period [12]. However, patients in the latter group more frequently experienced obstetric complications (fetal morbidity, miscarriages, premature births or intrauterine growth retardation) compared to APS patients associated with SLE. During the observation period, there was no difference in mortality between the two groups, and the most common causes of death were severe thrombotic events, including myocardial infarctions. Between the causes of death depending on the underlying disease, 10% of primary APS patients died of AMI. Among the 1000 patients in the cohort, 9 developed CAPS, and among these, 5 died (mortality 55.6%) [12].

A 2017 systematic review included 40 patients with AMI secondary to APS. AMI was the first presentation of APS in 80% of these cases. Cardiac catheterization showed normal coronaries in 75% of patients, whereas 25% revealed obstructive atherosclerotic stenosis [8].

**CAPS.** Definite CAPS is defined as all four criteria (Table 1). The pathophysiology of CAPS is distinguished by rapid microvascular thrombosis leading to widespread ischemic injury. The most recognized theory illustrates the role of uncontrolled complement activation with an identifiable precipitating event such as infection, surgery or pregnancy [13,14]. The data suggest different mechanisms, such as increased oxidative stress on the surface of the vascular endothelium, linked to the presence of aPLs that activate complement, leading to thrombosis [15]. Furthermore, numerous reports have suggested significant correlations between aCL and anti-β2GPI levels and the incidence and severity of acute coronary syndrome, as occurred in our patient [16,17].

Data from the International CAPS Registry and other studies have suggested that 50–53% of patients presenting with CAPS will have cardiac manifestations [18,19,20]; the heart is the fourth most commonly affected organ system. Typical evidence of cardiac disease in CAPS includes heart failure and valvular disease [4]. However, a recent study identified myocardial ischemia as the leading cause of cardiac manifestation; Troponin and NT-pro-BNP levels were elevated in 93% and 100% of patients, respectively [20]. There have been prior reports of presentation with AMI and cardiogenic shock, requiring extracorporeal membrane oxygenation (ECMO) [21]. Sadly, this syndrome can lead to a terrible prognosis, with a contemporary estimate of mortality of 33–37% [22,23]. While the primary cause of death in CAPS is cerebral death, cardiac causes are the second most common. The hallmark of autopsy in all cases is the presence of micro infarcts in multiple organ systems [22].

Gόmez-Puerta et al. reported that CAPS during the third trimester of pregnancy or puerperium represents only 6% of all cases of CAPS, and half of the patients had a history of APS [24,25]. In addition, compared to the general population with CAPS, those in pregnancy or puerperium contract CAPS at an earlier age and have fewer previous clinical manifestations [2]. 

Using the data derived from the literature, Table 3 summarizes the clinical manifestations, treatment given and maternal/fetal outcomes of each case of CAPS with cardiac involvement from 1987 until 2023, in chronological order.

Fetal death is the precipitating factor in 8% of cases in the CAPS registry [18]; in the cases listed in Table 3, there were 50% fetal deaths.

## 4. Discussion

CAPS is a rare form of APS that develops quickly, with multiorgan involvement and a high mortality rate, and its approach often poses a multidisciplinary challenge.

In the first week of breastfeeding, our patient developed multiple arterial thrombosis and clinical manifestations suggestive of CAPS; luckily, she had a healthy baby, but she risked having very serious complications.

The initial diagnosis of CAPS was clinical, given the sudden onset of thrombosis in large and small vessels, and across multiple organs, as clearly shown on angio-CT. Her livedo reticularis and thrombocytopenia confirmed previously diagnosed APS, and the initial trigger might have been the pregnancy that culminated in AMI and coma after a few days. 

As CAPS affects <1% of patients with APS, recommendations are based on case reports and expert opinions/consensus, and there are no randomized clinical trials to guide the diagnostics and treatment of CAPS [19,40,41]. 

CAPS continues to be a diagnostic challenge (due to its low prevalence) with a wide range of clinical signs, symptoms and laboratory findings, as seen in our case, that frequently overlap with other obstetric complications. Since there are few cases of pregnant or new mothers with CAPS and cardiac involvement, the implications related to diagnostics and therapy to be carried out during pregnancy are delicate and complex.

Its multisymptomatic nature, due to the involvement of multiple organs in CAPS, can highlight slightly elevated troponin, which can be misleading. If there is no confirmation of cardiac involvement through ECG, echocardiography or coronary angiography, it is useful not to stop in cases of elevated troponin. It is better to perform a cardiac MRI during the diagnostic process to demonstrate the microangiopathic process, as highlighted in our case.

Echocardiography and coronary artery (CA) angiography could be suggestive of small vessel infarcts, as CMRI more accurately evaluates the location and extent of microinfarcts. Furthermore, given that the diagnosis of CAPS requires involvement in multiple organ systems, CMRI is probably a tool that is not used often enough, even though it reaffirms the pathophysiology of CAPS and could lead clinicians to the possibility of a diffuse thrombotic process [42]. 

The potentially remitting course of cardiac involvement in CAPS dictates a strict follow-up with non-invasive techniques, such as CMRI, which allows for a re-evaluation of clinical and instrumental parameters, a crucial point for the appropriate management of these patients.

Myocardial damage in APS could even be subclinical, as suggested by the extremely high rate of fortuitous evidence of myocardial scarring in aPL-positive patients. Ischemic-type LGE at MRI can be demonstrated in 11% of APS patients, compared to 3.7% in controls [43].

These evidences account for the choice of not proceeding with endomyocardial biopsy in our patient, but rather to follow a rational and practical approach.

Presently, no single clinical or imaging finding exists to confirm the diagnosis, but an integrated approach including clinical history and assessment, lab tests and CMRI should be pursued. However, further studies are required to clearly ascertain this hypothesis. 

Our assembled team of neuro-cardio-rheumatology professionals suspected CAPS, which was quickly developing with multi-organ and life-threatening involvement. The increase in TnI, altered mental state, pulmonary/hepatic involvement and cutaneous manifestations suggested thrombotic microangiopathy from CAPS [44].

Another noteworthy issue concerns the early detection of cardiac involvement due to the high rate of mortality of CAPS (48%), mostly depending on cardiac problems [45]. Echocardiography and CA angiography are useful to find heart valve disease, heart dysfunction and coronary disease. Nevertheless, cardiac involvement may be presented as microvascular thrombosis or micro-vessel disease, sometimes without evident cardiac wall motion abnormalities. 

Similarly to our case, CMRI represents a non-invasive, non-radiating imaging technique that can identify and quantify both acute and old myocardial scarring by micro-vascular thrombosis. An ischemic pattern of fibrosis was revealed as a bright signal from LGE by PSIR T1 between 10 and 20 min after the contrast agent administration [46]. The ischemic transmural scar, the rise of TnI and symptoms of myocardial ischemia are signs of myocardial infarction with non-obstructive coronary arteries (MINOCA) that have been seen to be related to thrombophilia, including APS [47,48,49,50]. 

In our patient, MINOCA was a concern due to microthrombi that could have blocked the blood vessels. However, a diagnosis of CAPS was suspected after the heart catheterization was carried out and definitively confirmed with a CMRI.

Adenosine-stress myocardial perfusion-fibrosis CMRI has been noted to detect micro-vascular diseases, silent myocardial ischemia and fibrosis in APS in the absence of obstructive epicardial coronary arteries [51,52]. Small vessel diseases may have more diffuse deficit patterns, either circumferential or pure segmental perfusion defects. Compared to the coronary artery, small vessel diseases have a shorter time in terms of perfusion deficit in the first pass, and the contrast wash-in is sufficient. Only wash-out into subendocardial layers is limited. This method may exclude epicardial coronary stenosis and could be considered in follow-up, preventing the young woman from receiving pure diagnostic CA angiography [53]. Due to LGE on the middle lateral left ventricular wall, a stress-perfusion CMRI was not required for diagnosis. 

Finally, the loss of consciousness was not explained by organic defects in brain imaging, and the patient’s recovery may suggest a transitory ischemic attack (TIA). The high prevalence of heart and brain involvement with TIA or stroke in CAPS has given rise to the search for a possible application of combined brain/heart MRI, especially in the follow-up of high-risk patients [54,55].

In our center, CMRI is performed only twice a week; stress-perfusion CMRI and combined brain/heart MRI are not yet implemented, requiring more data and expertise. 

At the moment, no precise indications for CMRI in pregnant CAPS patients are described, but MRI is recommended if other non-invasive diagnostic measures are not sufficient for a definitive diagnosis, and is better than ionizing radiation-based imaging modalities when possible [56,57]. Although gadolinium-based contrast in the first trimester of pregnancy should be avoided, recent studies have examined the safety related to prenatal exposure to gadolinium-based contrast agents used in MRI and the risk thereof for fetal and neonatal death [58].

It is noteworthy that most of the patients in the post-partum or post-abortum period had a suspension of low molecular weight heparin (LMWH) for a few days associated with a withdrawal of aspirin, like our patient [32]. This could have facilitated the occurrence of CAPS.

Perhaps our case could have been better identified from the start. Even the aGAPSS score suggested by some authors may also have been a helpful tool in assessing the patient’s thrombotic risk and in making the best choice of antithrombotic therapy [59].

Collict et al. proposed a therapeutic strategy for CAPS during pregnancy and puerperium: (A) prevent and treat any trigger factor, providing any supportive care for any critically ill patient; (B) evaluate fetal lung maturation before delivery; and (C) use a combination of anticoagulation and immunosuppression despite the increased risk of bleeding [8,40]. CAPS is in fact a highly thrombotic state, despite its prolonged bleeding time and low platelets. 

“Triple therapy” is certainly the most accepted treatment to date and consists of anticoagulation, corticotherapy and therapeutic plasma exchange (TPE/plasmapheresis) or intravenous immune globulin (IVIG). New therapies, such as rituximab and eculizumab, may have a role as a second-line therapy in the treatment of complicated, refractory/relapsing CAPS patients [8,40].

Rapid deterioration can occur at any time in CAPS, causing harm to both mother and fetus. Proper management is therefore crucial for these patients. Our case is not the first, and it will not be the last, but early and accurate diagnosis with aggressive treatment will be able to reduce maternal and fetal mortality.

Physicians should be very cautious when a patient with APS reveals HELLP syndrome, and we are convinced that anticoagulants should be resumed in the first days of the post-partum period, even in those patients with severe thrombocytopenia secondary to HELLP syndrome.

Optimal management is linked with a reduction in maternal mortality, but fetal and neonatal prognoses depend mostly on the gestational age at the onset of CAPS. Subsequent pregnancies should be discouraged, and if they do occur, they should be carefully monitored. It is important that physicians counsel patients accordingly about their potential risks.

Therefore, pregnant patients known to have aPLs should be carefully monitored in the post-partum period for early signs or symptoms of pleuropolmonary or cardiac disease or thrombotic episodes.

According to the International Consensus statement on classification criteria, the diagnosis of CAPS was certain and further supported by clinical patients’ improvements with plasma exchange and therapy with intravenous immune globulin [3,45,60,61].

The prescribed dose of enoxaparin after childbirth was mistakenly 4000 IU per day, and was therefore too low for a case of APS with previous deep vein thrombosis. Inadequate anticoagulation and/or low INR may be precipitating factors for CAPS (8% of all causes) [45].

Secondary prevention with APS and a previous venous thromboembolic event requires LMWH and low-dose aspirin, and should be continued for at least six weeks following delivery [62,63]. As recommended by the American Society of Hematology and by the McMaster RARE-Bestpractices project group, we decided to adjust LMWH therapy and to add antiplatelet therapy, taking into account from the very beginning the high risk of vascular events [61]. For the first-line treatment of patients with CAPS, a combination therapy with glucocorticoid, heparin and plasmapheresis or IVIG is suggested over single therapy or other combinations, with significant lower mortality [62]. Moreover, the components of the combination may be given stronger consideration based on clinical subsets of CAPS. In our case, with thrombotic microangiopathy, we decided to start with plasmapheresis over intravenous immune globulin (IVIG). 

## 5. Conclusions

We aimed to report this case to shed light on the management of cardiovascular complications in pregnant patients with CAPS and report on published cases.

CAPS with cardiac involvement is a rare and life-threatening form of APS rarely seen in pregnancy, making early recognition difficult. Any pregnant woman who presents multi-organ thrombosis, thrombocytopenia and positive aPLs, albeit without the classic cardiovascular risk factors, should be considered with suspicion. 

CMRI could detect scarring LGE caused by micro-vascular thrombosis through PSIR T1, CA and micro-vessel diseases through the stress-perfusion technique. 

This case describes a rare complication of CAPS, stressing the importance of early and timely recognition, daily multidisciplinary evaluation and the initiation of aggressive and immediate treatment [61,64].

We hope we have provided an important message for clinicians not to stop the diagnostic search even if the troponin I level is slightly elevated. Otherwise, the diagnosis of cardiac involvement (which sometimes may be presented without evident cardiac symptoms) may be missed, and precious time for the timely initiation of therapy may be lost.

The present report is only the third study that has demonstrated, with the use of CMRI, the detailed microvascular involvement of the myocardium in a young living mother with CAPS, the youngest described in the literature in this context [33,38]. From retrospective studies, there is still an underuse of CMRI in APS patients (30% of cases), especially in pregnancy, but when this is performed, it is always pathological [19]. CAPS is a clinical emergency, and clinicians need to be aware that timely diagnosis and treatment may improve both maternal and fetal outcomes.

## Figures and Tables

**Table 1:** Criteria for the classification of catastrophic APS.

Criteria for CAPS	
1. Evidence of involvement of three or more organs, systems and/or tissues. Usually, clinical evidence of vessel occlusions, confirmed by imaging techniques when appropriate. Renal involvement is defined by a 50% rise in serum creatinine, severe systemic hypertension (>180/100 mmHg) and/or proteinuria (>500 mg/24 h).	
2. Development of manifestations simultaneously or in less than a week.	
3. Confirmation by histopathology of small vessel occlusion in at least one organ or tissue. For histopathological confirmation, significant evidence of thrombosis must be present, although vasculitis may coexist occasionally.	
4. Laboratory confirmation of the presence of antiphospholipid antibodies (lupus anticoagulant and/or anticardiolipin antibodies). Antiphospholipid antibodies must be detected on two or more occasions at least six weeks apart (not necessarily at the time of the event).	
**Diagnosis of CAPS**	
Definite CAPS	All 4 criteria.
Probable CAPS	All 4 criteria, except for only two organs, systems and/or tissues involvement.
	All 4 criteria, except for the absence of laboratory confirmation at least six weeks apart due to the early death of a patient never tested for aPL before the catastrophic APS.
	1, 2 and 4.
	1, 3 and 4 and the development of a third event in more than a week but less than a month, despite anticoagulation.

aPL: anti-phospholipid antibodies; APS: antiphospholipid syndrome; CAPS: catastrophic antiphospholipid syndrome. Note. Adapted with permission from Ref. [3]. 2003, Sage.

**Table 2:** Clinical laboratory data of CAPS in the young patient.

Organ Manifestation	Clinical Signs	Altered Laboratory Data	(Reference Range)	Normal Laboratory Tests
**Lung** Bilateral Pulmonary Embolism	Polypnea Dyspnea	Hemoglobin: 79 g/L WBC 16 × 10^9^/L Platelet count: 75 × 10^9^/L	110–153 g/L 3.5–11.0 × 10^9^/L 115–370 × 10^9^/L	
**Kidney**	-	Proteinuria >500 mg/g Creat. Leukocyturia 596 cel/µL Erythrocyturia 343 cel/µL	<80 Negative Negative	Serum creatinine
**Brain**	Headache Blurred vision Neurosensory deficit Vomit attacks Comatose state	pH 7.47 pCO_2_ 34 pO_2_ 72 HCO_3_^−^ 21	7.35–7.45 35–45 mmHg 80–100 mmHg 22–26 mmol/L	Absence of Leiden Factor V mutation Absence of prothrombin mutation PT20210A Protein C and protein S
**Heart** MINOCA	Epigastric pain Chest pain Hypotension Tachycardia	I-Troponin (HS) 4741 ng/L	<40 ng/L	NT-proBNP
**Skin**	Fever Livedo reticularis			Anti-mitochondria antibody (AMA) Anti-smooth muscle antibody (ASMA)
**Liver** Elevated liver enzymes	Right hypochondrium pain	AST 70 U/L ALT 88 U/L	0–35 U/L 0–35 U/L	Bilirubin (total and direct) GammaGT Anti-Liver-Kidney Microsomial antibody
**Pancreas**	-	Pancreatic amylase 67 U/L LDH 365 U/L	0–53 U/L 0–210 U/L	Anti-gastric parietal cells antibodies
**Spleen**	-	D-dimers 5850 μg/L	0–500 μg/L FEU	
**Thyroid**	-			TSH
**Placenta**	Areas of necrosis Vascular congestion Small placental infarctions	Fibrinogen 574 mg/dL	200–400 mg/dL	INR: 1.1 aPTT: 36
**Infections**		VES 79 mm/h	0–30	Procalcitonin 0.22 µg/L (<0.50)
		C-Reactive Protein 12.3 mg/dL	0–0.50	Anti-HIV 1–2 (CHIV ag/Ab), Ag-HBs, Anti-HCV, SARS-CoV-2
				Blood culture (×8): negative
				Urine culture (×3): negative
				Rectal swab: negative
		Anti-HSV 1-2 IgG antibodies Anti-EBV antibodies Anti-OMV antibodies	Present Present Present	Toxoplasmosis, Varicella-Zoster virus, Cytomegalovirus, Measles antibodies, Paramyxovirus, Echovirus, Coxachievirus, Adenovirus, hRSV, Chlamydia pneumoniae, Q-Fever, Ab. Anti T. pallidum Ig
**Immune system**		Anti-Double-Stranded-DNA antibodies 65 UI/mL	≤35 UI/mL	Rheumatoid factor
		LAC 1.36/2.35	Ratio < 1.20	Functional Antithrombin III
		Anti-Cardiolipin IgG 851 U/mL Anti-Cardiolipin IgM 770 U/mL	<20 <20	Complement 3 1.5 g/L (0.9–1.8)
		aβ2GPI IgG 2761 U/mL aβ2GPI IgM >840 U/mL	<20 <20	Complement 4 0.16 g/L (0.10–0–40)
		ANA 1:160	<1:80	IgG, IgM, IgA (mg/dL): negative

aβ2GPI: anti-β-2-glycoprotein I; Ag-HBs: antigen-Hepatitis B surface; ALT: alanine aminotransferase; ANA: antinuclear antibodies; Anti-EBV: anti-Epstein-Barr virus antibodies; Anti-HCV: antibodies-Hepatitis C virus; Anti-HIV 1-2: antibodies-Human immunodeficiency virus type1-2; Anti-OMV: anti-orthomyxovirus antibodies; Anti-HSV: anti-herpes simplex virus antibodies; aPTT: activated partial thromboplastin time; AST: aspartate aminotransferase; CHIV ag/Ab: HIV antigen/antibody combo assay; FEU: Fibrinogen Equivalent Units; GammaGT: gamma-glutamyl transferase; HIV: human immunodeficiency virus; hRSV: human respiratory syncytial virus; INR: International Normalized Ratio; LAC: Lupus anticoagulant (two tests); LDH: lactate dehydrogenase; NT-proBNP: N-terminal proBrain Natriuretic Peptide; PT20210A: prothrombin 20210A; SARS-CoV-2: severe acute respiratory syndrome Coronavirus 2; TSH: thyroid-stimulating hormone; VES: velocity of Erythrocyte Sedimentation; WBC: white blood cell.

**Table 3:** Twenty-one cases of CAPS involving the heart in pregnancy and the puerperium.

Author/Year	DOI/PMID	Maternal Age	Gestational Age (Time of Onset)	CAPS Features	Treatment	Maternal Follow-Up	Fetal Outcome
Bendon RW et al., 1987 [26]	https://pubmed.ncbi.nlm.nih.gov/3827544 PMID: 3827544	22 years	30 week of gestation	Cardiac, renal, placenta, gastrointestinal and myometrium TMA		Death	Intrauterine fetal death
Hochfeld M et al., 1994 [27]	https://pubmed.ncbi.nlm.nih.gov/8159355/ PMID: 8159355	37 years	Second day after fetal death	Cardiac, neurological, renal failure, pulmonary, splenic, adrenal infarct, cerebral haemorrhage	Cyclophosphamide, S, plasma exchange	Death	Intrauterine fetal death
Ortiz P et al., 2003 [28]	https://pubmed.ncbi.nlm.nih.gov/14658174/ PMID: 14658174	32 years	32 week of gestation	Cardiac (aortic valve lesions), renal, neurological, thrombocitopenia	A, H, S	1 years	Healthy twins
Coward LJ et al., 2005 [29]	https://doi.org/10.1136/jnnp.2005.066746 PMID: 16227567	30 years	3rd week of puerperium	Cardiac, cerebral, renal, pulmonary, hepatic, adrenal haemorrhage	Inotropic support, hemofiltration	Death	Healthy child
Ciolkiewicz M et al., 2006 [30]	https://pubmed.ncbi.nlm.nih.gov/16780270/ PMID: 16780270	24 years	30th day of puerperium	Cardiac, pulmonary, renal, multiorgan failure	H, S, IVIG, plasma exchange	Death at 1 month	Fetal death
Zieba B et al., 2009 [31]	https://pubmed.ncbi.nlm.nih.gov/19650000/ PMID: 19650000	29 years	18th day of puerperium	Cardiac, renal, cerebral, thrombocytopenia	A, S, IVIG, IABP, plasmapheresis	3 months	Healthy child
Hanouna G et al., 2013 [32]	https://doi.org/10.1093/rheumatology/ket167 PMID: 23676524						
(Case 2)		32 years	8th day of puerperium	Cardiac, renal, hepatic, cutaneous, HA, thrombocytopenia	H, A, S, IVIG	2.3 years	Healthy twins
(Case 3)		26 years	25 week of gestation	Cardiac, neurological, renal, cutaneous, HA	H, S, IVIG, plasma exchange, dialysis	5.8 years	Fetal death
(Case 4)		31 years	Third day of puerperium	Cardiac, renal, splenic, cutaneous, hepatic, thrombocytopenia, HA	H, A, S, IVIG, dialysis	3.9 years	Fetal death
(Case 7)		32 years	Fourth week of puerperium	Cardiac, neurological, pulmonary, renal, hepatic, pancreatic, splenic, ocular, thrombocytopenia	H, S, IVIG, plasma exchange, dialysis	8 years	Fetal death
(Case 8)		29 years	15th day of puerperium	Cardiac, neurological, renal, cutaneous, hepatic, pancreatic, gastric, ocular, thrombocytopenia, HA	H, S, plasma exchange, dialysis	Sudden death at 6 years	Fetal death
(Case 10)		36 years	Third day of puerperium	Cardiac, neurological, renal, hepatic, pancreatic, cutaneous, thrombocytopenia, HA	H, S	5 years	Healthy child
(Case 12)		27 years	On the day of delivery at 13 weeks gestation	Cardiac, cutaneous, hepatic, placenta, thrombocytopenia	H, S, IVIG	5.2 years	Fetal death
(Case 13)		23 years	31 week of gestation	Cardiac, renal, cutaneous, thrombocytopenia, HA	H, A, S, plasma exchange	Sudden death at 2.5 years	Child with developmental delay
Rijo D et al., 2017 [33]	https://pubmed.ncbi.nlm.nih.gov/29898299 PMID: 29898299	32 years	15th day of puerperium	Cardiac, cerebral, multiorgan failure	IVIG, S	N/A	N/A
Khizroeva J et al., 2019 [34]	https://doi.org/10.1080/14767058.2017.1422715 PMID: 29284338	24 years	28 week of gestation	Cardiac, pulmonary, placenta, thrombocytopenia	H, S, ultrafiltration	2 years	Prematurity
Ruffatti A et al., 2019 [35]	https://doi.org/10.1016/j.autrev.2019.03.015 PMID: 30844561	32 years	29 week of gestation	Cardiac, pulmonary, renal	H, S, IVIG, plasma exchange, ECMO, ecolizumab, Bi-Vad	1 years	Healthy child
Schultz M et al., 2019 [36] (case 2)	https://doi.org/10.1177/0961203319871099 PMID: 31451079	37 years	N/A	Cardiac, renal, small vessel thromboembolic disease.	H, S, plasmapheresis, rituximab, W	N/A	Fetal death
Pinto V et al., 2021 [37]	https://doi.org/10.1186/s12959-021-00356-w PMID: 34930339	38 years	32 week of gestation	Cardiac, renal, pulmonary, hepatic, splenic, arterial-venous thrombosis	H, plasmapheresis, rituximab, vitamin K antagonist	Six months	Fetal death
Rato IR et al., 2021 [38]	https://doi.org/10.1177/09612033211002273 PMID: 33736518	32 years	39th week of gestation	Cardiac, cerebral, placenta	S, H, IVIG,	2 years	Healthy child
Collict M et al., 2021 [39]	https://doi.org/10.1136/bcr-2019-230863 PMID: 31527209	31 years	28 week of gestation	Cardiac, cerebral, placenta, pulmonary, splenic	S, IVIG, H, A, W	8 months	Healthy child

A: aspirin; Bi-Vad: extra-body biventricular assist device; ECMO: extra-Corporeal Membrane Oxygenation; H: heparin; HA: haemolytic anaemia; IABP: intra-aortic balloon pump; IVIG: intravenous immunoglobulin; N/A: not available; S: steroids; TMA: thrombotic microangiopathy; W: warfarin.

## Data Availability

Data are available on reasonable request to the corresponding author.
